# Clinical Evaluation of Metagenomic Next-Generation Sequencing for the detection of pathogens in BALF in severe community acquired pneumonia

**DOI:** 10.1186/s13052-023-01431-w

**Published:** 2023-02-18

**Authors:** Yupeng Wei, Tongqiang Zhang, Yuting Ma, Jisi Yan, Jianghua Zhan, Jun Zheng, Yongsheng Xu

**Affiliations:** 1 Clinical School of Paediatrics, Tianjin Medical University, Tianjin, China; 2 Department of Respiratory Medicine, Tianjin Children’s Hospital (Tianjin University Children’s Hospital), Tianjin, China; 3 Department of Neonatology, Tianjin Central Hospital of Gynecology Obstetrics, Tianjin, China; 4 Department of Infection, Tianjin Children’s Hospital (Tianjin University Children’s Hospital), Tianjin, China; 5 Department of Pediatric Surgery, Tianjin Children’s Hospital (Tianjin University Children’s Hospital), Tianjin, China

**Keywords:** Metagenomic next-generation sequencing (mNGS), Serious community acquired pneumonia (SCAP), Bronchoalveolar lavage fluid (BALF), Children, Pathogen

## Abstract

**Background:**

Rapid and accurate identification of pathogens is very important for the treatment of Severe community-acquired pneumonia (SCAP) in children. Metagenomic Next-generation sequencing (mNGS) has been applied in the detection of pathogenic bacteria in recent years, while the overall evaluation the application of SCAP in children is lacking.

**Methods:**

In our study, 84 cases of SCAP were enrolled. Bronchoalveolar lavage fluid (BALF) samples were analysed using mNGS; and sputum, blood, and BALF samples were analysed using conventional technology (CT).

**Results:**

Among the 84 children, 41 were boys, and 43 were girls, with an average age ranging from 2 months to 14 years. The pathogen detection rate of mNGS was higher than that of CT (83.3% [70/84] vs. 63.1% [53/84], P = 0.003). The mNGS was much greater than that of the CT in detecting Streptococcus pneumoniae (89.2% [25/29] vs. 44.8% [13/29], P = 0.001) and Haemophilus influenzae (91.7% [11/12] vs. 33.3% [4/12], P < 0.005). The mNGS also showed superior fungal detection performance compared with that of the CT (81.8% [9/11] vs. 18.2% [2/11], P = 0.004). The mNGS test can detect viruses, such as bocavirus, rhinovirus, and human metapneumovirus, which are not frequently recognised using CT. However, the mNGS detection rate was lower than that of the CT (52.4% [11/21] vs. 95.2% [20/21], P = 0.004) for Mycoplasma pneumoniae (MP). The detection rate of mNGS for mixed infection was greater than that of the CT, although statistical significance was not observed (26.3% [20/39] vs. 21.1% [16/39], P > 0.005). Treatment for 26 (31.0%) children was changed based on mNGS results, and their symptoms were reduced; nine patients had their antibiotic modified, five had antibiotics added, nine had their antifungal medication, and seven had their antiviral medication.

**Conclusion:**

mNGS has unique advantages in the detection of SCAP pathogens in children, especially S. pneumoniae, H. influenzae, and fungi. However, the detection rate of MP using mNGS was lower than that of the CT. Additionally, mNGS can detect pathogens that are not generally covered by CT, which is extremely important for the modification of the treatment strategy.

## Background

Community-acquired pneumonia (CAP) is a common respiratory disease in children with significantly higher morbidity and mortality rates in developing countries than that in developed countries [1]. According to the World Health Organization (WHO) report, the incidence of CAP in children under 5 years of age in developing countries is 0.28 times/child/year, accounting for 95% of all cases of CAP in children worldwide, and the mortality rate is 1.3–2.6% [2]. In North America and Europe, the incidence rate among pre-schoolers is 36 per 1,000 children per year [3], placing a significant economic burden on families and society. Children with CAP are prone to develop pleural effusion, lung abscess, pericarditis, meningitis, and other pulmonary and external complications [4], which eventually lead to severe community-acquired pneumonia (SCAP), respiratory and circulatory failure, or even life-threatening complications. For the pathogen diagnosis of CAP, clinical work is carried out in various microorganisms, including viruses, bacteria, and atypical pathogen combination detection to avoid missed diagnosis and mixed infection, although pathogens cannot be identified or detected in 22–57% of children [5–7] and 13–62% of adult with pneumonia [8, 9]. Culture, the gold standard for bacterial and fungal infection diagnosis, has high accuracy but a long detection time and low detection rate and is not suitable for the identification of pathogens that are difficult to cultivate. Viruses and atypical pathogens can be detected by polymerase chain reaction (PCR) and antigen detection, but the positive rate varies greatly and suitable for detecting only the known, limited target pathogens. In addition, some unknown pathogens in nature cannot be detected. Therefore, there is an urgent need for rapid and accurate diagnostic methods to identify pathogens in children with SCAP [10].

Metagenomic next-generation sequencing (mNGS) is a method for whole nucleic acid detection using patient samples directly [11], which can simultaneously detect various microorganisms. It has the characteristics of being less affected by antibiotics, taking a wide range of samples, and does not need to set gene sequences in advance. Therefore, it greatly applicable in diagnosis of new and rare infectious diseases [10, 12–14]. Currently, mNGS has been applied in the diagnosis of infectious diseases of the lungs, blood system, and central nervous system in adults [15–17]. Sepsis, encephalitis, and bone and joint infections have also been reported in children [18–20]. However, no study has been conducted on bronchoalveolar lavage fluid (BALF) in children with SCAP. In this study, 84 children with SCAP were enrolled for bronchoscopic lavage. BALF was used as the sample source to evaluate the value of mNGS in the diagnosis of SCAP in children by comparing it with conventional technology (CT).

## Methods

### Patient Inclusions

In this retrospective analysis, 84 children diagnosed with SCAP were enrolled in Tianjin Children's Hospital from January 2019 to December 2021. After admission, a fiberoptic bronchoscopy was done within 48 h. bronchoalveolar lavage fluid (BALF) was taken for mNGS detection and CT was performed simultaneously.

**Fig. 1:**
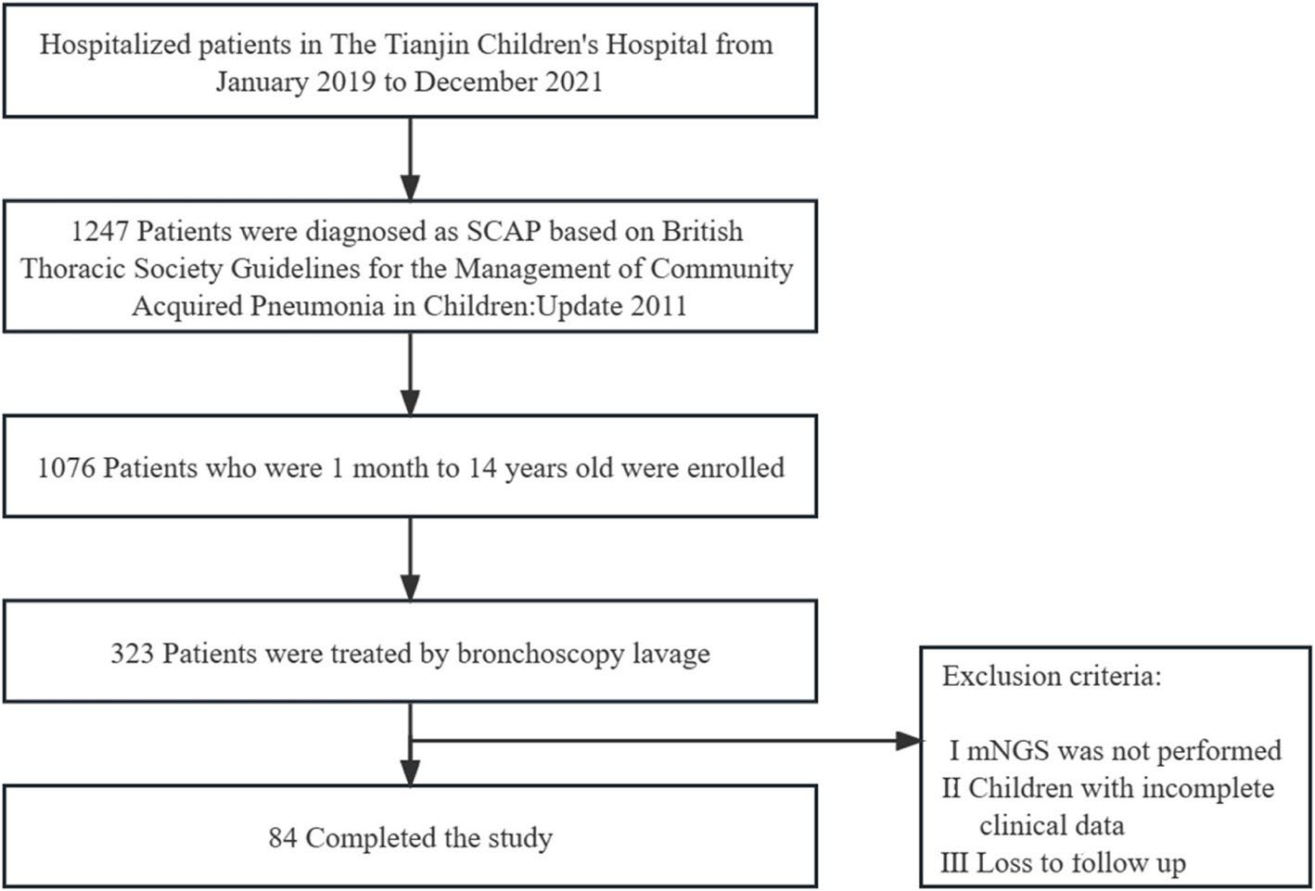
Flowcharts of enrollment and diagnosis

Inclusion criteria were as follows: (I) all patients met the SCAP diagnostic criteria; (II) pediatric patients under 14 years of age were selected; (III) BALF obtained by bronchoscopy in patients who met the bronchoscopy indications. The following conditions will be excluded from the study: (I) mNGS was not performed; (II) incomplete clinical data; (III) loss to follow up. The study was approved by The Tianjin Children’s Hospital Ethics Committee (No. L2020-02) and was in line with the Declaration of Helsinki (as revised in 2013) (Fig. 1).

SCAP definition (Includes one of the following): (I) oxygen saturation < 92%, cyanosis; (II) respiratory rate > 50 breaths/min, Infant respiratory rate > 70 breaths/min; (III) significant tachycardia for level of fever (values to define tachycardia vary with age and with temperature); (IV) prolonged central capillary refill time > 2 s; (V) difficulty in breathing; (VI) intermittent apnoea, grunting; (VII) signs of dehydration; (VIII) Infant feeding difficulties [21].

Indications for fiberoptic bronchoscopy: (I) repeated or persistent wheezing; (II) limited wheezing; (III) recurrent respiratory tract infections; (IV) hemoptysis; (V) Difficulty in withdrawing from the ventilator; (VI) Abnormal chest imaging: ① atelectasis, ② emphysema, ③ mediastinal emphysema, ④ pulmonary mass lesion, ⑤ pleural cavity lesion requiring differential diagnosis; (VII) Etiological diagnosis and treatment of pulmonary infectious diseases [22].

The study was approved by the ethics committee of Tianjin Children's Hospital, and patient data were analyzed anonymously.

### Conventional technology (CT)

For hospitalized children with SCAP, blood and sputum were collected within 24 h of admission, and alveolar lavage fluid was collected within 48 h of admission. The sample source for the CT technique is mostly the BALF because it is challenging to obtain sputum from children and blood infection is quite uncommon in this study. Bacteria was detected by culture method and commercial PCR-based kits for the detection eight common respiratory bacteria (*Streptococcus pneumoniae*, *Staphylococcus aureus*, *methicillin resistant Staphylococcus aureus*, *Klebsiella pneumoniae*, *Pseudomonas aeruginosa*,*Acinetobacter baumannii*, *Stenostomonas maltophilia*, and *Haemophilus influenzae*) (Capitalbio Jinxin Bio- Technology Co., Ltd., Chengdu, China). The virus was detected by PCR (*Epstein–Barr virus* (EBV), *cytomegalovirus* (CMV), *influenza A/B* (Da’an Gene Technology Co., 96 Ltd., Guangzhou, China); and *adenoviruses* (Puruikang Biotech Co., Ltd., Shenzhen, China)). Fungi (*Candida albicans*, *Pneumocystis vulgaris*, *Cryptococcus neoformans*) was detected by culture method. *Mycobacterium tuberculosis* was detected by culture, PCR and MTB-Xpert. PCR was used to detect *chlamydia* and *Legionella*. *Mycoplasma pneumoniae* was detected by particle agglutination (PA) (serum MP-Ab ≥ 1:160 or MP-Ab increased or decreased by 4 times or more in recovery and acute stages), immune colloidal gold technigue (GICT) (single MP-IgM positive) [23] and PCR.

### mNGS testing

#### Sample processing and nucleic acid extraction

The BALF sample, which is viscous and contains particles in suspension, is liquefied. Utilized a DTT reagent with a concentration of 80 mM. According to the viscosity of the sample, 1 ~ 3 times of the sample volume of DTT was added, and the sample was shaken and left at room temperature for five minutes. Non-viscous samples were directly enriched by centrifugation and the samples were stored at -20 °C. DNA was extracted from all samples using a QIAamp® UCP Pathogen DNA Kit (Qiagen) following the manufacturer’s instructions. Human DNA was removed using Benzonase (Qiagen) and Tween20 (Sigma). Total RNA was extracted with a QIAamp® Viral RNA Kit (Qiagen) and ribosomal RNA was removed by a Ribo-Zero rRNA Removal Kit (Illumina). cDNA was generated using reverse transcriptase and dNTPs (Thermo Fisher).

#### Library construction and sequencing

Libraries were constructed for the DNA and cDNA samples using a Nextera XT DNA Library Prep Kit (Illumina, San Diego, CA). DNA library construction includes fragmentation, end repair, ligation and purification of connectors, PCR amplification and purification, library concentration, and fragment quality monitoring. For effective libraries, the Qubit concentration was at least 1 ng/L, and the primary peak of the library fragment was between 280 and 320 bp. Library was quality assessed by Qubit dsDNA HS Assay kit followed by High Sensitivity DNA kit (Agilent) on an Agilent 2100 Bioanalyzer. Library pools were then loaded onto an Illumina Nextseq CN500 sequencer for 75 cycles of single-end sequencing to generate approximately 20 million reads for each library. For negative controls, we also prepared PBMC samples with 105 cells/mL from healthy donors in parallel with each batch, using the same protocol, and sterile deionized water was extracted alongside the specimens to serve as non-template controls (NTC).

#### Bioinformatics analyses

Trimmomatic was used to remove low quality reads, adapter contamination, and duplicate reads, as well as those shorter than 50 bp. Low complexity reads were removed by Kcomplexity with default parameters. Human sequence data were identified and excluded by mapping to a human reference genome (hg38) using Burrows-Wheeler Aligner software. We designed a set of criteria similar to the National Center for Biotechnology Information (NCBI) criteria for selecting representative assembly for microorganisms (bacteria, viruses, fungi, protozoa, and other multicellular eukaryotic pathogens) from the NCBI Nucleotide and Genome databases. The final database consisted of about 13,000 genomes. Microbial reads were aligned to database with SNAP v1.0beta.18. The database performance (a certain number of clinical validation samples) has a specificity of 96.2% and a sensitivity of 100%.

#### Interpretation of mNGS results

Currently, there is a lack of unified interpretation standards for mNGS. This study formulated the following standards based on previous literature [15, 24–26].

1) When the relative abundance of an organism at the species level is greater than 30% and there is supporting literature evidence of lung pathogenicity, regardless of the results of the CT method.

2) mNGS detected the same pathogenic bacteria as CT and more than 50 single microbe reads.

3) *Mycobacterium tuberculosis* and *Legionella pneumophila* are considered positive when at least one read is mapped to the species or genus level.

4) Oral and respiratory custom bacteria are not considered pathogenic bacteria, regardless of their relative abundance, unless otherwise proved or they are considered pathogenic bacteria.

CT and NGS alone could not determine whether the microorganism was infected. Finally, two senior attending physicians determined the pathogen based on clinical characteristics and reached a consensus.

### Statistical analysis

Data were processed using SPSS 26.0. Continuous variables were expressed as mean ± standard deviation (SD) and assessed by independent group t tests. Categorical variables were expressed as percentage (%) and assessed by Chi-squared tests or Fisher’s exact test. A two-sided α less than 0.05 were considered as statistically significant.

## Results

### General characteristics of selected children

Among the 84 patients, 41 were boys, and 43 were girls, with age ranging from 2 months to 14 years and an average of 4.4 years. There were 18 (21.4%) children with basic diseases, 6 (7.1%) had congenital heart disease; 4 (4.8%) had bronchial asthma; and primary ciliary dyskinesia, hepatoblastic carcinoma, renal malformation, giant gyri malformation, diabetes, chronic granuloma, pelvic tumour, and congenital cleft palate was each reported in 1 child. The most common symptoms were cough (92.9%), fever (73.8%), and dyspnoea (35.7%), followed by poor mental health (27.4%), stridor (26.2%), and chest pain (11.9%), and the last was haemoptysis (2.4%). A total of 65 cases (77.4%) reported moist rales, and 26 (31%) had diminished respiratory sounds. In addition, 54 (62.3%) children exhibited severe lung imaging, including 36 with pleurisy (42.9%), 28 with inflammatory consolidation (33.3%), 24 with (28.6%), 20 with pleural effusion (23.8%), 20 with atelectasis (23.8%), 11 with necrosis (13.1%), and 7 with interstitial abnormalities (8.3%). The white blood cell (WBC) count (12.57 ± 6.46 × 10^9^/L) was calculated, as was the neutrophil ratio (77.06 ± 11.96%), the lymphocyte ratio (13.98 ± 6.30%), the C-reactive protein (CRP) (77.13 ± 59.58 mg/L), and the procalcitonin (PCT) (11.03 ± 17.57 ng/mL). Before admission, 72 (85.7%) patients had been treated with antibiotics and 20 (23.8%) with glucocorticoids (Table 1).

**Table 1:** Clinical information of children

Clinical characteristics	
Characteristics	
Sex, male, *n* (%)	41 (48.8%)
Age, mean (range), years	4.4 (0.17–14)
Symptoms or signs	
Fever, *n* (%)	62 (73.8%)
Cough, *n* (%)	78 (92.9%)
Dyspnea, *n* (%)	30 (35.7%)
Chest pain, *n* (%)	10 (11.9%)
Hemoptysis, *n* (%)	2 (2.4%)
Mentally weak/poor, *n* (%)	23 (27.4%)
Whoop, *n* (%)	22 (26.2%)
Rale, *n* (%)	65 (77.4%)
Diminished respiration, *n* (%)	26 (31.0%)
Imaging features	
Consolidation ≥ 1/2 of the lung, *n* (%)	28 (33.3%)
Pulmonary atelectasis, *n* (%)	20 (23.8%)
Pleural effusion, *n* (%)	24 (28.6%)
Pleurisy, *n* (%)	36 (42.9%)
Pulmonary interstitial changes,* n* (%)	7 (8.3%)
Lung necrosis, *n* (%)	11 (13.1%)
Laboratory parameters	
White blood cell counts (109 /L) (mean ± sd)	12.57 ± 6.46
Lymphocyte [%] (mean ± sd)	13.98 ± 6.30
Neutrophil [%] (mean ± sd)	77.06 ± 11.96
C-reactive protein (mg/L) (mean ± sd)	77.13 ± 59.58
Procalcitonin (ng/mL) (mean ± sd)	11.03 ± 17.57
Basic disease	
Congenital heart disease, *n* (%)	6 (7.1%)
Primary ciliary dyskinesia, *n* (%)	1 (1.2%)
Asthma, *n* (%)	4 (4.8%)
Hepatoblastoma, *n* (%)	1 (1.2%)
Renal malformations, *n* (%)	1 (1.2%)
Giant gyrus malformation, *n* (%)	1 (1.2%)
Diabetes, *n* (%)	1 (1.2%)
Chronic granuloma, *n* (%)	1 (1.2%)
The pelvic neoplasm, *n* (%)	1 (1.2%)
Congenital cleft palate, *n* (%)	1 (1.2%)
Pre-admission interventions	
Antibiotics, n (%)	72. (85.7%)
Glucocorticoid, n (%)	20 (23.8%)

### The pathogen detection of SCAP

**Fig. 2:**
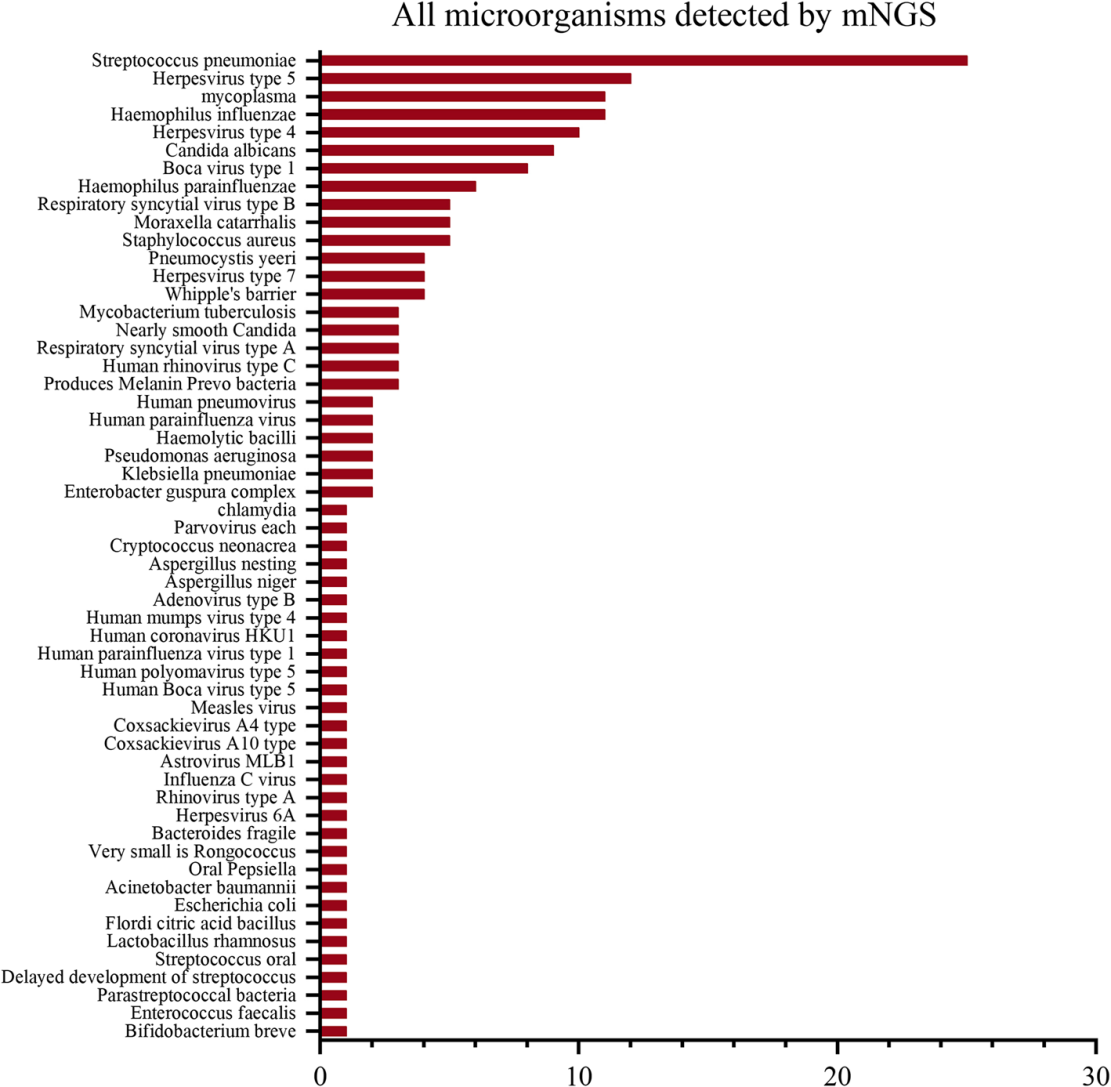
All microorganisms detected by Metagenomic Next-Generation Sequencing

The most common pathogen detected using mNGS was *Streptococcus pneumoniae* (25 strains), human herpesvirus type 5 (12 strains), *Haemophilus influenzae* (11 strains), and *Mycoplasma pneumoniae* (MP) (11 strains). Pathogens, such as oral *Streptococcus*, *Bifidobacterium brevis*, *Bacteroides fragilis*, and *Candida proximalis*, were also detected. Combined with clinical manifestations, the bacteria were considered as respiratory colonising bacteria, not pathogenic ones (Fig. 2).

The most common pathogens detected using CT were MP (20 strains), *S. pneumoniae* (13 strains), and *Staphylococcus aureus* (10 strains).

**Fig. 3:**
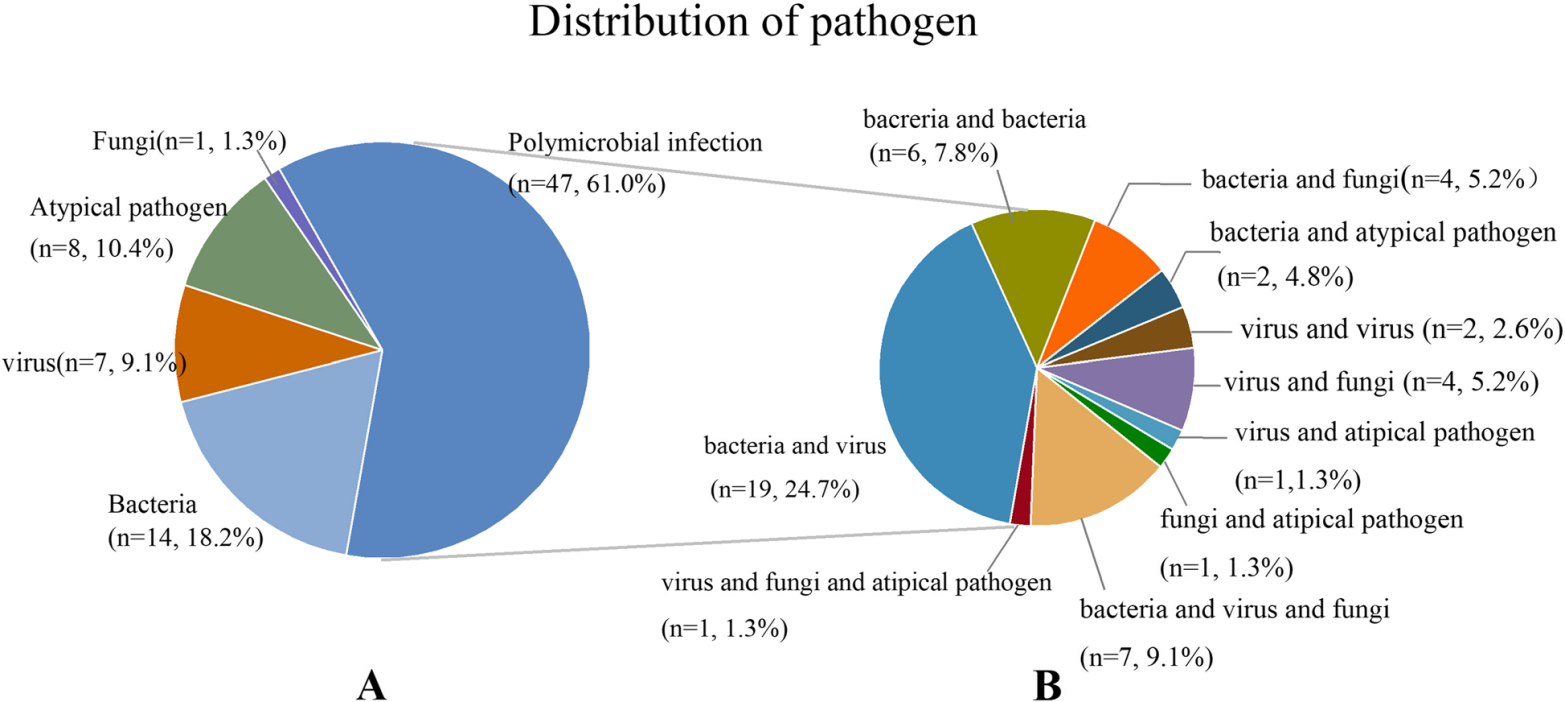
**A** Metagenomic Next-generation sequencing combined Conventional technology detected the pathogen classification. **B** Metagenomic Next-generation sequencing combined Conventional technology diagnose the type of polymicrobial infection

Finally, 76 cases (90.5%) were diagnosed with determinate pathogen infection, and 18 (23.7%) were bacterial infections. Six patients (7.9%) were infected with a virus, and there were 10 (13.2%) cases of atypical bacterial infection. In addition, there were three (3.9%) cases of *Mycobacterium tuberculosis* (MTB) infection and 39 (51.3%) of mixed infection (Fig. 3A).

### The detection rate of mNGS and CT

The sensitivity, specificity, positive predictive value and negative predictive value of mNGS were 92.1% (70/76), 62.5% (5/8), 95.9% (70/73) and 45.5% (5/11), respectively. mNGS detection rate was 83.3% (70/84), which was higher than that of the CT at 63.1% (53/84) (*P* = 0.003).

**Fig. 4:**
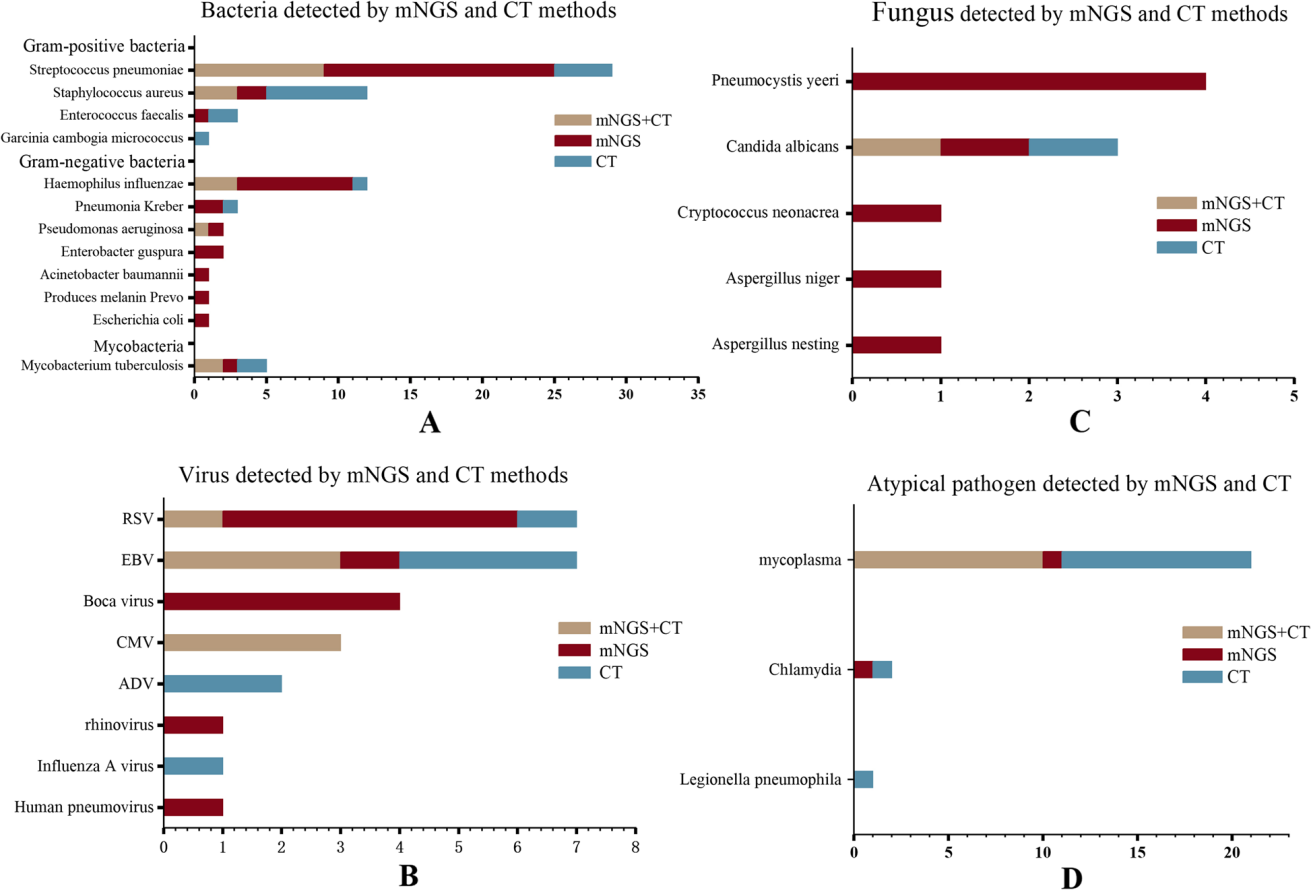
Comparison of pathogens detected by Metagenomic Next-generation sequencing (mNGS) and Conventional technology (CT). **A** Bateria detected by mNGS and CT methods. **B** Virus detected by mNGS and CT methods. **C** Fungus detected by mNGS and CT methods. **D** Atypical pathogen detected by mNGS and CT methods. ADV, adenovirus; CMV, human herpes virus; EBV, Epstein-Barr virus,; RSV, Respiratory syncytial virus

The bacterial detection rate of mNGS was higher than that of the CT (80.8% [42/52] vs. 44.2% [23/52], *P* < 0.001) (Fig. 4A). The detection rate of Gram-negative bacteria was significantly higher in mNGS than that in the CT (90.1% [20/22] vs. 22.7% [5/22], *P* < 0.001), while there was no significant difference in Gram-positive bacteria (75% [30/40] vs. 57.5% [23/40], *P* = 0.098). *S. pneumoniae* was the most detected bacteria in 29 cases, in which the detection rate of mNGS was higher than that of the CT (89.2% [25/29] vs. 44.8% (13/29), *P* = 0.001). A total of nine cases were detected using mNGS and CT, sixteen were only positive for mNGS, and four were only positive for CT. The second most detected bacteria were *H. influenzae* (12 cases), in which the detection rate of mNGS was higher than that of the CT (91.7% [11/12] vs. 33.3% [4/12], *P* < 0.005). mNGS alone detected eight cases, CT alone detected one, and common method detected three. There were 12 cases of *S. aureus*, three were detected using mNGS and CT, two were only positive for mNGS, and seven were positive for CT. Among the three children with *Enterococcus faecalis* infection, two were detected using CT and one using mNGS. *E. faecium* and *Micrococcus gamboge* were identified by culture but not by mNGS in patient 59. There were three cases of *Klebsiella pneumoniae*, one in patient 11, one in patient 44, detected using mNGS, and one in patient 64, detected using CT. There were two cases of *Pseudomonas aeruginosa*, one of which was detected using both mNGS and CT and the other using mNGS alone. Additionally, mNGS detected *Prevotella melanogenes* in patient 26, *Enterobacter cloacae* in patients 43 and 44, *Escherichia coli* in patient 50, and *Acinetobacter baumannii* in patient 65, all of which were not detected on CT.

For virus detection, the detection rate of mNGS was higher than that of the CT, but there was no statistical significance (76% [19/25] vs. 56% [14/25], *P* = 0.136) (Fig. 4B). *Respiratory syncytial virus* (RSV) and *Epstein-Barr virus* (EBV) were the most frequently detected viruses, with seven cases each. mNGS alone identified five cases of RSV, while CT alone identified one case; however in patient 79, RSV was detected using both mNGS and CT. EBV was detected by CT in six cases (patients 9,10, 13, 53, 63, and 65), and by mNGS in four cases (patients 13, 36, 63, and 65). *Boca virus* (HBoV) was detected in four cases, all of which were detected by mNGS. In three cases of CMV infection, both mNGS and CT were positive. Two cases of *adenovirus* (ADV) and one case of *influenza A virus* were detected using CT. However, mNGS detected *bocavirus* in patients 39, 42, 68, and 76, *rhinovirus* in patient 58, and *human metapulmonary* virus in patient 66.

The detection rate of fungi using mNGS was higher than that of the CT (81.8% [9/11] vs. 18.2% [2/11], *P* = 0.004) (Fig. 4C). *Pneumocystis vulgaris* (four cases) was the most frequently detected fungus, followed by *C. albicans* (three cases). There was one case of *Cryptococcus neoformans*, *Aspergillus aspergillus*, and *Aspergillus niger*. Only two instances of *C. albicans* were discovered using CT in patients 62 and 83. However, mNGS missed only patient 83. In patient 26, mNGS and CT were both negative, but the serum G test (1, 3-D glucan test) was positive (301 pg/mL), which matched the clinical symptoms and imaging diagnosis of fungal infection.

There was no significant difference in the detection rate of MTB between mNGS and CT (60% [3/5] vs. 80% [4/5], *P* > 0.005). A total of two cases were detected using mNGS and CT, one case was detected using mNGS alone, two cases were detected using CT, three were detected using MTB-Xpert in CT, and one by culture method (Table 2).

**Table 2:** The detection of M. tuberculosis by mNGS and CT

	Patient no	mNGS		culture	MTB-Xpert
**M. tuberculosis**	02	+	-		-
	06	-	-		+
	07	-	-		+
	14	+	-		+
	32	+	+		-

*mNGS* Metagenomic Next-generation sequencing, *CT* Conventional technology

Among atypical bacterial infections, mNGS detection rate was lower than that of the CT (50.0% [12/24] vs. 91.7% [22/24], *P* = 0.003) (Fig. 4D). CT found one case of *Legionella pneumophila* and one of *Chlamydia trachomatis* alone. The detection rate of MP using mNGS was lower than that of the CT (52.4% [11/21] vs. 95.2% [20/21], *P* = 0.004). In addition, 10 cases were positive for both mNGS and CT, one for mNGS alone, and 10 for CT alone. CT included MP-polymerase chain reaction (PCR) detection in 57.1% (12/21), PA detection in 52.4% (11/21), and GICT detection in 14.3% (3/21) (Table 3).

**Table 3:** The detection of MP by mNGS and CT

	Patient no	mNGS	PA	PCR	GICT
**MP**	01	+	+	+	-
	03	+	+	+	-
	04	+	-	+	-
	07	-	-	+	-
	09	+	+	+	-
	10	+	-	+	-
	12	+	+	+	-
	13	-	-	+	-
	15	+	-	+	-
	16	+	+	-	-
	18	+	+	-	-
	19	+	+	+	+
	22	+	-	-	-
	24	-	-	+	-
	27	-	+	-	-
	29	-	+	-	-
	30	-	-	-	+
	41	-	-	-	+
	57	-	-	+	-
	63	-	+	-	-
	83	-	+	-	-

*mNGS* Metagenomic Next-generation sequencing, *CT* Conventional technology, *GICT* immune colloidal gold technigue, *PCR* Polymerase Chain Reaction, *PA* particle agglutination, *MP* Mycoplasma, MP infection can be confirmed when serum MP-Ab ≥ 1:160 is used as an indicator of recent infection, or MP-Ab is 4 times higher or lower in recovery and acute stage. Single detection of MP-IgM positive by GICT is valuable for the diagnosis of MP infection

### The mixed infection of SCAP

There were 39 (51.3%) cases of mixed infection. CT alone detected 16 (21.1%) cases of mixed infection, and mNGS alone detected 20 (26.3%) cases of mixed infection. However, this difference was not statistically significant (*P* > 0.005).

Mixed bacterial and viral infections were the most common type, with 12 (30.8%) cases. Eight (20.5%) patients had mixed bacterial and fungal infections and five (12.8%) cases of mixed bacterial and atypical pathogens (Fig. 3B).

### Diagnosis consistency between mNGS and CT

A total of 49 (58.3%) cases were positive for both mNGS and CT, 22 (26.2%) for only mNGS, 5 (6.0%) for only CT, and 8 (9.5%) were negative for both CT and mNGS.

**Fig. 5:**
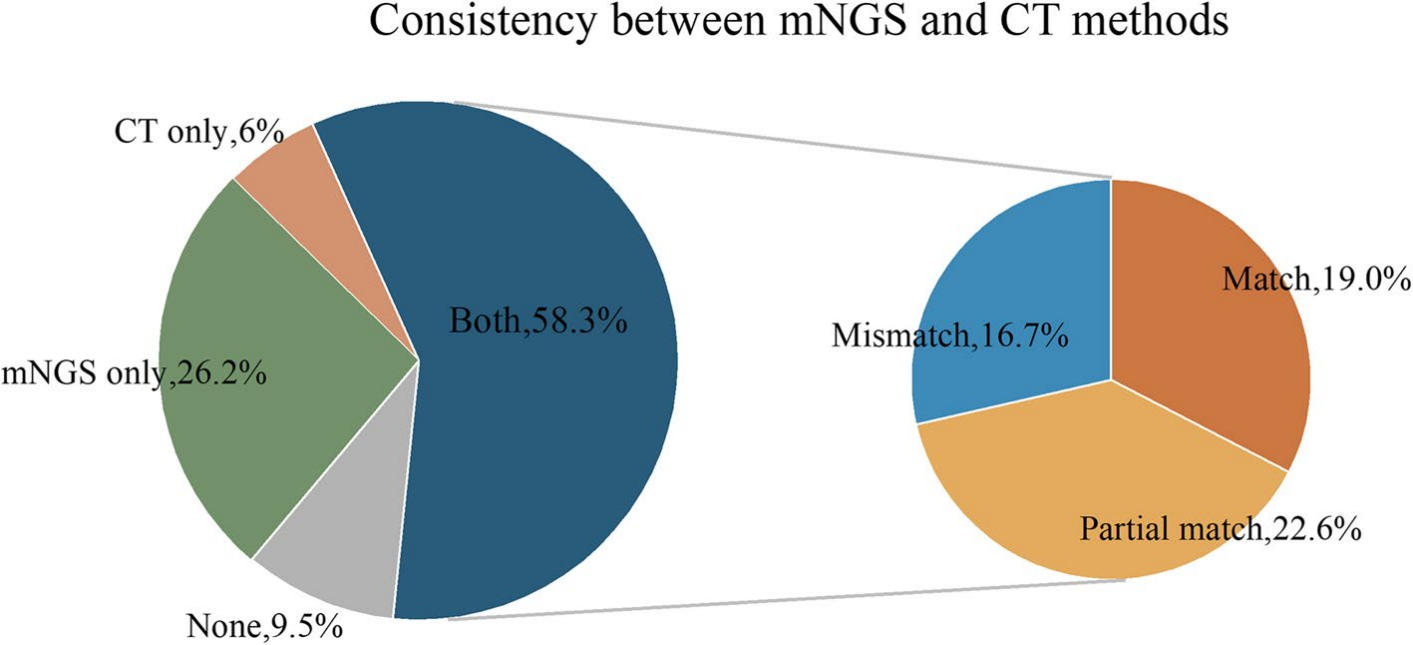
Consistency between Metagenomic Next-generation sequencing and Conventional technology diagnosis

Among 49 double-positive cases, mNGS and CT results were completely consistent in 16 (19.0%) patients and completely inconsistent in 14 (16.7%) cases. Nineteen (22.6%) cases were partially consistent, indicating that at least one detected pathogen overlapped between CT and mNGS (Fig. 5).

### Changes after the detection of mNGS

There were 26 (31.0%) patients who changed their treatment based on mNGS results, and their symptoms were reduced. Antibiotics were changed in nine patients, antibiotics were added in five patients, antifungal drugs in nine, antivirals in seven, and antituberculosis drugs in one (Table 4).

**Table 4:** Changes in treatment of children with mNGS

Patient no	mNGS-based diagnosis	Change the treatment
02	Mycobacterium tuberculosis	Add anti tuberculosis drugs
05	Streptococcus pneumoniae Haemophilus influenzae Cryptococcus neoformans Proximate smooth candida	Add Fluconazole
07	Staphylococcus aureus	Latamoxef Sodium change to Linezolid
11	Streptococcus pneumoniae Klebsiella pneumoniae Pneumocystis yersini	Latamoxef Sodium change to Linezolid and add Sulfamethoxazole
19	Streptococcus pneumoniae MP	Latamoxef Sodium change to Linezolid
22	Streptococcus pneumoniae Pneumocystis yersini MP	Add Sulfamethoxazole and Azithromycin
25	Streptococcus pneumoniae Haemophilus influenzae	Latamoxef Sodium Chane to Piperacillin and Tazobactam
31	Streptococcus pneumoniae	Latamoxef Sodium Change to Ertapenem
36	Streptococcus pneumoniae EBV	Add Duffy
39	Bocavirus	Add Duffy
42	Boca virus	Add Duffy
43	Enterobacter cloacae	Add Cefoperazone
44	Klebsiella pneumoniae Enterobacter cloacae	Add Cefoperazone
45	Haemophilus influenzae Candida albicans	Add Fluconazole
49	Streptococcus pneumoniae	Latamoxef Sodium Change to Linezolid
50	Escherichia coli	Latamoxef Sodium Change to Cefoperazone
53	Excrement enterococcus Pseudomonas aeruginosa	Add Linezolid
58	Rhinovirus Construct nests aspergillus	Add Fluconazole and Duffy
60	Haemophilus influenzae RSV	Latamoxef Sodium Change to Cefoperazone and add Duffy
62	Staphylococcus aureus Candida albicans	Latamoxef Sodium Change to Linezolid and add Fluconazole
68	Bocavirus	Add Duffy
70	Pneumocystis vulgaris	Add Sulfamethoxazole
72	Aspergillus Niger	Add Fluconazole
76	Bocavirus	Add Duffy
77	Chlamydia	Add Etoerythromycin
82	Pneumocystis vulgaris	Add Sulfamethoxazole

*mNGS* Metagenomic Next-generation sequencing, *CT* Conventional technology, *MP* Mycoplasma, *EBV* Epstein-Barr virus, *RSV* Respiratory syncytial virus, *ADV* Adenovirus, *MRSA* Methicillin resistant Staphylococcus aureus

## Discussion

Our study comprehensively evaluated the application of mNGS in children with SCAP. In this study, pathogen detection rate of mNGS was higher than that of the CT (83.3% vs. 63.1%, *P* = 0.003), especially for *S. pneumoniae*, *H. influenzae*, and fungi. There was no significant difference between mNGS and CT in the detection of tuberculosis and virus. However, MP detection rate was lower for mNGS than that for CT (52.4% [11/21] vs. 95.2% [20/21], *P* = 0.004). In addition, mNGS can detect pathogens that cannot be detected using CT, which is of great significance for the adjustment of antimicrobial therapy.

BALF was used as the sample source in this study because it was collected from infected sites and was less contaminated by oral colonisation bacteria [27, 28]. Regarding bacterial detection, our study found that mNGS had a higher detection rate for *S. pneumoniae* than that of the CT (89.2% vs. 44.8%, *P* = 0.001). In a study on children with CAP by Farnaes et al., 6.7% (1/15) of *S. pneumoniae* were detected by culture and cell-free plasma next-generation sequencing (CFPNGS); pneumococcus was only detected using CFPNGS in the other eight cases [29]. There was another study on invasive pneumococcal disease in 96 children; positive rate of culture method was 27.1% (*n* = 26), while that of mNGS test was 62.5% (*n* = 60) (blood, cerebrospinal fluid, and pleural effusion samples) [30]. These findings suggest that when considering *S. pneumoniae* infection in children with SCAP, mNGS should be actively performed to clarify the aetiology and guide anti-infection treatment. In this study, the detection of *S. aureus* using mNGS was lower than that of traditional methods (41.7% vs. 83.3%). However, Ren et al.'s study collected noted that mNGS (blood, cerebrospinal fluid, and BALF) was more sensitive than that of the culture method (77.8% vs. 44.4%) in adult patients with sepsis [24]. We utilised both culture and PCR, which may have enhanced the rate of *S. aureus* identification by conventional methods. This may also be associated with the different immune systems of children and adults.

For Gram-negative bacteria, our study found that the mNGS detection rate was significantly higher than that of the CT, especially for *H. influenzae* (91.7% vs. 33.3%, *P* < 0.005). mNGS also had a greater detection rate for *P. aeruginosa*, *K. pneumoniae*, and *E. coli* than that of the CT in our study. The study by Wu et al. on adult SCAP also revealed that the rate of *H. influenzae* identification using mNGS was higher than that of the conventional detection techniques (100% vs. 33.3%) in BALF [31]. In a study on adult sepsis, Ren et al. discovered that mNGS had a greater detection, n rate of *P. aeruginosa* and *K. pneumoniae* than that of the culture method (75.4% vs. 43.5%, 81.6% vs. 26.3%, respectively), using blood, cerebrospinal fluid, and BALF samples [24]. According to the aforementioned study, mNGS should be actively performed whenever Gram-negative bacterial infection is considered. However, a previous study revealed that after paediatric haematopoietic stem cell transplantation, the detection rate of *P. aeruginosa* and *K. pneumoniae* using conventional methods is comparable to that of the mNGS using BALF [32]. These differences may be due to the different specimen sources and immune functions.

Regarding virus detection, mNGS showed no difference compared with that of the CT in the detection of ADV, EBV, and influenza A viruses in this study. When children with SCAP caused by the above pathogens are clinically suspected, traditional methods should be considered first for pathogen detection. A previous study showed that mNGS has a low sensitivity for influenza A viruses [33]. Real-time fluorescence quantitative PCR is more sensitive than mNGS for the detection of influenza virus infection in adult SCAP [34]. In another study, mNGS (RNA-SEQ) did not detect any of the eight positive ADV samples for paediatric pneumonia but had high sensitivity to rhinovirus and RSV (100% and 96%, respectively) [35]. The potential of mNGS to identify additional viruses and subtypes that cannot be detected by conventional methods has also been confirmed in previous studies [33, 36]. In contrast, mNGS assisted in identifying cytomegalovirus (CMV) infection and not merely colonisation. In our study, mNGS and CT eventually detected three children with CMV infection, including one patient with congenital heart disease. Based on the findings of the mNGS, two cases were treated with antiviral medications, and the patients were cured. In a study, one child with leukaemia received antiviral treatment after CMV was detected using mNGS, and their symptoms improved [37]. This demonstrates that CMV should be appraised not only based on colonisation but also on the child’s underlying illness and clinical characteristics.

The fungal detection rate of mNGS was significantly higher than that of CT alone. In a study on the detection of fungal pneumonia in adults, the culture method was only used in 1/21 samples, while mNGS was used in 19/21 samples [38]. In a study of children after haematopoietic stem cell transplantation, mNGS (BALF) detected nine cases of fungal pneumonia (*Pneumocystis jiroi* [*n* = 6], Aspergillus [*n* = 2], and mucor [*n* = 1]), while none was detected using traditional methods [32]. In this study, the positive rate of fungal pneumonia detected using mNGS was 81.8% (9/11), while only 18.2% (2/11) used the traditional pathogen detection method. Among them, 54.5% (6/11) had underlying diseases, including congenital heart disease, primary ciliary dyskinesia, chronic granuloma, pelvic mass, diabetes, and congenital cleft palate. Two kinds of *Aspergillus, Aspergillus aspergillus* and *Aspergillus niger*, were also detected using mNGS, but not by traditional methods. Huang et al. also reported that mNGS detected various *A. fumigatus, A. niger, A. flavus, and A. oryzae* in immunosuppressed adult patients diagnosed with fungal pneumonia [39]. Therefore, mNGS should be actively applied to children with severe pneumonia and underlying diseases or immunodeficiency. In our study, three cases of *C. albicans* infection were confirmed, and the detection rates of mNGS and CT were similar, among which two cases had underlying diseases. Liu et al. also reported that *C. albicans* was one of the fungi most frequently detected in BALF cultures [40]. However, another study found that the mNGS detection rate of *C. albicans* was lower than that of the traditional method (70.6% vs. 82.4%) in ventilators-associated pneumonia in adults [25]. Therefore, traditional methods should be used for pathogen detection in children with *C. albicans* infection. Interestingly, patient 26 tested negative on mNGS and CT but positive on the 1,3-D glucan test, which combined clinical symptoms and imaging to diagnose fungal infection. Therefore, culture, mNGS, galactomannan (GM) test, and 1, 3-D glucan test should be combined in the diagnosis of fungal pneumonia to avoid missing positive samples.

The detection sensitivity of mNGS for MTB was similar to that of CT in our study. Previous studies have also confirmed that the diagnostic ability of mNGS for MTB is similar to that of the traditional detection methods [41, 42]. Therefore, when clinically considering the presence of MTB infection, traditional methods rather than mNGS should be considered first. MTB, a type of intracellular bacteria, releases less nucleic acid into the extracellular environment, and insufficient wall fracture can also affect the extraction of DNA; therefore, fewer sequences can be detected [43, 44]. mNGS combined with MTB-Xpert can be used to improve diagnostic rates [11, 43].

Surprisingly, we found that mNGS was inferior to CT in the detection of atypical pathogens (50.0% vs. 91.7%), especially for MP (52.4% vs. 95.2%). This has never been mentioned in previous studies. Only studies on children with severe unresponsive pneumonia indicated that mycoplasma detection rates of mNGS and PCR were equal (15.6% vs. 18.8%) [37]. In this study, the detection rate of MP-PCR was 57.1% (12/21), which was similar to that by mNGS. The possible reasons are the combination of PCR and two immunological methods, which increased the sensitivity of the traditional method in this study. Therefore, when considering MP infections in children with SCAP, it is not necessary to conduct mNGS examinations.

The patient received empirical anti-infective medication in the absence of knowledge of its precise aetiology. After identifying the illness' pathogenic microorganisms, 26 people (31.0%) modified their course of treatment according to the mNGS results, and their symptoms improved. In a study of adults with severe respiratory diseases, mNGS led to a change in treatment in 34.4% (11/32) of patients [13]. Combining mNGS with conventional detection technologies can improve the ability to target drug use. On the other hand, the culture process takes at least 3 days [45], while mNGS can obtain test results within 24–48 h [36]. Although PCR is quick, its reach is constrained and may not even detect the causal agent [33]. These results suggest that mNGS is important for the detection and treatment of SCAP in children.

Our study has several limitations. First, the sample size of our study was modest; hence, the number of pathogens identified was limited. Second, because of the high sensitivity of mNGS, pathogens of colonisation and contamination will also be discovered; therefore, there is no universal standard for the report's issuance and interpretation. Finally, because this was a single-centre observational study, a case selection bias may have occurred. Further prospective, multicentre, randomised controlled studies are needed to confirm the sensitivity, specificity, and clinical value of mNGS.

## Conclusion

In conclusion, mNGS has its unique advantages in the detection of pathogenic agents of SCAP in children, especially Streptococcus pneumoniae, Haemophilus influenzae and fungi. In addition, mNGS can detect pathogens that cannot be detected by CT, which is of great significance for adjusting antimicrobial therapy.

## Data Availability

The raw sequence data reported in this paper have been deposited in the Genome Sequence Archive (Genomics, Proteomics & Bioinformatics 2021) in National Genomics Data Center (Nucleic Acids Res 2022), China National Center for Bioinformation / Beijing Institute of Genomics, Chinese Academy Sciences (GSA: CRA008578) that are publicly accessible at https://ngdc.cncb.ac.cn/gsa.

## Abbreviations

CAP: Community-acquired pneumonia
SCAP: Severe community-acquired pneumonia
mNGS: Metagenomic next-generation sequencing
BALF: Bronchoalveolar lavage fluid
CT: Conventional technology
MP: Mycoplasma pneumoniae
EBV: Epstein–Barr virus
CMV: Cytomegalovirus
RSV: Respiratory syncytial virus
ADV: Adenovirus
MRSA: Methicillin resistant Staphylococcus aureus
MTB: Mycobacterium tuberculosis
PCR: Polymerase chain reaction
PA: Particle agglutination
GICT: Immune colloidal gold technigue
